# 基于质谱的单细胞蛋白质组学分析方法及应用

**DOI:** 10.3724/SP.J.1123.2020.08030

**Published:** 2021-02-08

**Authors:** Shaojie QIN, Yu BAI, Huwei LIU

**Affiliations:** 北京大学化学与分子工程学院, 北京分子科学国家研究中心, 北京 100871; College of Chemistry and Molecular Engineering, Peking University, Beijing National Laboratory for Molecular Sciences, Beijing 100871, China; 北京大学化学与分子工程学院, 北京分子科学国家研究中心, 北京 100871; College of Chemistry and Molecular Engineering, Peking University, Beijing National Laboratory for Molecular Sciences, Beijing 100871, China; 北京大学化学与分子工程学院, 北京分子科学国家研究中心, 北京 100871; College of Chemistry and Molecular Engineering, Peking University, Beijing National Laboratory for Molecular Sciences, Beijing 100871, China

**Keywords:** 毛细管电泳, 液相色谱, 质谱, 微流控, 蛋白质组学, 单细胞, 综述, capillary electrophoresis (CE), liquid chromatography (LC), mass spectrometry (MS), microfluidic, proteomics, single cell, review

## Abstract

细胞是生命体的最小组成单位,遗传及外部环境等因素使单细胞异质性广泛存在于众多生物体中。传统的生物学实验获得的结果多是大量细胞的平均测量值,因此在单细胞层面开展研究对于精确理解细胞的生长发育以及疾病的诊断与治疗至关重要。而作为重要的细胞和生命活动的执行者,蛋白质由于其不具备扩增特性,且种类繁多、丰度低、动态分布范围宽,与核酸等其他生物大分子相比,其单细胞组学研究相对滞后。而在所有的检测手段中,荧光检测以及电化学分析方法具有极高的灵敏度,但是囿于其研究通量有限,以及电化学活性依赖,很难成为普适性的单细胞蛋白质组学研究方法。质谱分析作为传统蛋白质组学中最为核心的研究技术,由于其高灵敏、高通量、结构信息丰富等特点,在单细胞蛋白质组学研究中独树一帜。该文综述了近年来基于质谱的单细胞蛋白质组学研究中的代表性方法,根据质谱分析前蛋白质分离方式的差异,将其分为基于毛细管电泳分离、液相色谱分离和无分离手段的直接检测3类方法,在介绍研究现状的同时对这些方法在细胞通量、蛋白质鉴定数目、灵敏度以及方法应用方面进行了总结与比较。最后,基于目前研究中面临的挑战以及发展趋势对基于质谱的单细胞蛋白质组学的研究前景进行了展望。

## Abstract (en)

The cell is the smallest unit of living organisms. Although cells often assemble to serve a common function, intercellular heterogeneity often exists due to different genetic and environmental effects. Therefore, single-cell analysis has been regarded as an indispensable means to investigate cell heterogeneity, especially when researching cell differentiation, disease diagnosis, and therapy. As the chief factors influencing cell and biological activities, proteins have long been a major concern in biochemistry. However, due to their intrinsic lack of amplification characteristics, wide species variety, low abundance, and wide dynamic range, proteins are scarcely studied in single-cell research when compared with other biological macromolecules. Therefore, ultra-sensitive single-cell proteomics analysis methods are urgently required. Among all general measurement techniques, fluorescence methods possess high sensitivity and a capability of dynamic tracing, but low target numbers impose restrictions on their broad application in real “proteomic” studies. Similarly, electrochemical methods adapt to electrochemically active molecules, which miss the majority of proteins. Mass spectrometry (MS), as the core approach of proteomic studies, provides high-sensitivity and high-throughput analysis of proteins together with abundant structural information, which is unique in all the analytical instruments and has made great progress in single-cell proteomic research. Herein, the representative research methods for single-cell proteomics based on MS are reviewed. According to the different protein separation methods used prior to MS analysis, they are divided into three categories, including capillary electrophoresis (CE), liquid chromatography (LC), and direct infusion without the need for separation. First, CE has been widely used in the separation and analysis of complex biological samples owing to its low cost, high analysis speed, and high separation efficiency. Its unique feature is the extraction and transfer of contents from cellular or subcellular regions using capillaries smaller than a single cell size. This sampling method also offers less substrate interference and negligible oxidative damage to the cells. Nonetheless, single-cell analysis based on CE-MS mainly focuses on proteomic studies of large cells because of the considerable sample loss, interface instability, and reproducibility issues. Compared with CE, LC, especially nanoLC, is more widely used in single-cell proteomic research, which mainly depends on its good reproducibility, nanoliter injection volume, low flow rate, low sample loss, and good compatibility with mass spectrometry. In recent years, it has been increasingly applied in the study of large-volume embryos, germ cells, and even somatic cells. More than 1000 proteins have been identified in single HeLa cells using this state-of-the-art single-cell proteomics method. It is worth noting that the single-cell sampling volume based on LC gradually reduces to the nanoliter level, and that the sample loss can be reduced by integrating a series of proteomic sampling processes into small volumes, setting sealing conditions, and reducing washing steps. However, the adequacy of cell lysis, the completeness and efficiency of protein pretreatment, and the labeling of peptide segments are important factors affecting the number and types of protein identification. Compared with protein separation using CE or LC prior to MS analysis, the direct MS analysis, assisted by labelling and signal transformation, eliminates complicated sample pretreatment and simplifies the operation by reducing enzymatic hydrolysis and separation. It also renders higher resolution as well as multi-omics compatibility. So far, the number of proteins detected using this method is limited due to the complexity of the samples. In conclusion, the aspects of throughput, sensitivity, identified protein species, and applications are summarized for each method mentioned above, and the prospect of single-cell proteomic research based on MS in the future is also discussed.

细胞是构成生物体的基本单位^[1]^,由于遗传因素、生化噪音^[2]^、细胞微环境^[3]^等诸多因素使得单个细胞之间存在着广泛的异质性。因此,单细胞研究不仅使人类对细胞与生命的本质有更为精确的认识,也为疾病的诊断、分型、治疗以及预后提供了更为强有力的工具^[4,5]^。蛋白质作为生命活动的主要承担者,可以为其提供更为直接且更有价值的表型信息,因此成为单细胞研究的热点目标。单细胞内蛋白质种类繁多,丰度低,动态分布范围宽^[6]^且无法扩增,因此对检测手段提出了更高的灵敏度要求。在众多高灵敏分析方法中,荧光检测方法的灵敏度可以达到单分子水平^[7]^,并且具有动态跟踪能力^[8]^,但是其蛋白质检测通量有限。然而电化学检测方法虽然细胞干扰小^[9]^,但受限于蛋白质通量^[10]^以及电化学活性^[11]^的要求,无法对多种蛋白质进行同时检测。质谱作为研究蛋白质组学的一种常规方法,其灵敏度高,通过已有的蛋白质数据库,可以同时对上万种蛋白质进行定性以及定量,并且可以提供丰富的蛋白质结构信息。但是由于单个体细胞内的蛋白质总量平均只有约100 pg^[6]^,利用质谱直接进行检测会面临样本复杂度和单细胞灵敏度等挑战,因此质谱前的分离技术对于提高方法检测灵敏度以及蛋白质定性定量的准确度来说十分必要。目前,基于质谱的单细胞蛋白质组学研究方面的综述仍较少^[6,12-16]^,并且以分离方式为主线进行梳理的还未见报道。本文综述了近几年基于质谱的具有代表性的单细胞蛋白质组学研究方法及其应用。根据质谱分析前分离技术的差异,我们从基于毛细管电泳-质谱(CE-MS)、液相色谱-质谱(LC-MS)以及无分离的直接检测模式等3方面进行介绍,并从细胞以及蛋白质分析通量,方法灵敏度,蛋白质来源及其丰度,以及方法的应用等角度对上述方法进行总结与比较。

## 1 基于毛细管电泳的分离

毛细管电泳因其成本较低,分析速度快、分离效率高,已广泛应用于复杂生物样本的分离分析^[17]^。CE具有nL级进样量,可直接在细胞或组织中进行微区取样,从而避免基质干扰^[18]^、氧化损伤^[19]^等,基于CE-MS的单细胞蛋白质组学分析研究及应用开展较早。2014年,Sun等^[20]^借助超灵敏的电驱动鞘液型接口将毛细管区带电泳与串联质谱相结合,从300 ng Hela细胞的蛋白酶解液中鉴定到2100种蛋白质,并对牛血清白蛋白的酶解液中添加的血管紧张肽Ⅱ进行检测^[21]^,检出限可以低至2 amol,相对标准偏差值小于4%。与少量细胞或者少量蛋白酶解液的分析相比,对单个细胞中内源蛋白质的分析更能反映细胞的状态和功能。受限于方法的灵敏度,因此人们首先尝试在较大的单细胞中利用CE-MS进行内源蛋白质的分析,其中最具代表性的就是Nemes教授课题组的工作。该课题组^[22]^首先将目光瞄准经典的16-细胞非洲爪蟾早期胚胎的囊胚细胞(见图1a)。利用显微解剖的方法从胚胎中分离得到单细胞,之后依次经历细胞裂解、蛋白质还原与烷基化,以及过夜酶解等蛋白质组学前处理流程,最后通过毛细管电泳-微升电喷雾-高分辨质谱(CE-μESI-HRMS)对蛋白质进行定性以及定量分析,最终从单个囊胚细胞(直径约150 μm)的20 ng非卵黄蛋白中鉴定到了1630种蛋白质。通过比较不同发育阶段的胚胎细胞的蛋白质组鉴定结果,发现在胚胎的转录程序尚未开始的发育阶段早期,细胞沿着胚胎的多个体轴的翻译模式具有显著的异质性,该结果为之前报道的单细胞转录组测序的结果^[23]^提供了补充。毛细管由于内径小于囊胚细胞的大小(见图1b),因此在光学显微镜的指导下,可以利用毛细管对16-细胞胚胎特定区域进行采样,取样速度不仅更快,也提高了空间分辨率。Lombard-Banek等^[19]^对胚胎进行亚细胞区域采样后,提取物在压力作用下转移至微管中进行蛋白质酶解。进样10 nL酶解液后,利用毛细管电泳-纳升电喷雾-高分辨质谱分析,得到低至700 zmol的检出限,并在5 ng蛋白酶解物中鉴定到约800种蛋白质。利用该方法分析胚胎细胞向神经组织细胞分裂分化过程中的蛋白质组变化。结果表明,与显微解剖方法相比,微采样的方法耗样少、采样流程简化,且由于基质干扰的大大降低使蛋白质鉴定能力显著增强。除此之外,该课题组还对体积略小一些的单个活斑马鱼胚胎进行了研究,通过控制肽段流出速度使之与质谱的数据采集周期相匹配,进一步提高了肽段的分析和鉴定能力,研究结果也证实了不同类型的胚胎细胞间的表达异质性。而在CE前加入预分离过程,可进一步提高蛋白质鉴定数量。Choi等^[24]^通过在CE前加入用于预分离的反相C18微柱,对单细胞水平下的老鼠海马体神经元的蛋白质酶解物进行了分析(见图1c),最终从500 pg蛋白酶解物中鉴定到141个蛋白质。虽然这一灵敏度已接近单细胞水平,但样本复杂度相比单个细胞仍较低。此外,其他高灵敏分析技术也被用于单细胞检测,Geng等^[25]^将毛细管电渗驱动与激光诱导荧光技术相结合,实现了单个Hela细胞中Her2蛋白的原位检测。类似的,Chen等^[26]^利用可以透膜的荧光探针实现单个单核巨噬细胞内半胱氨酸组织蛋白酶家族的标记与检测。尽管借助荧光的检测技术灵敏度明显提高,但是蛋白质通量仍较低,远未达到组学的研究需要。因此发展基于毛细管电泳的高通量质谱方法仍然十分必要。

**图1:**
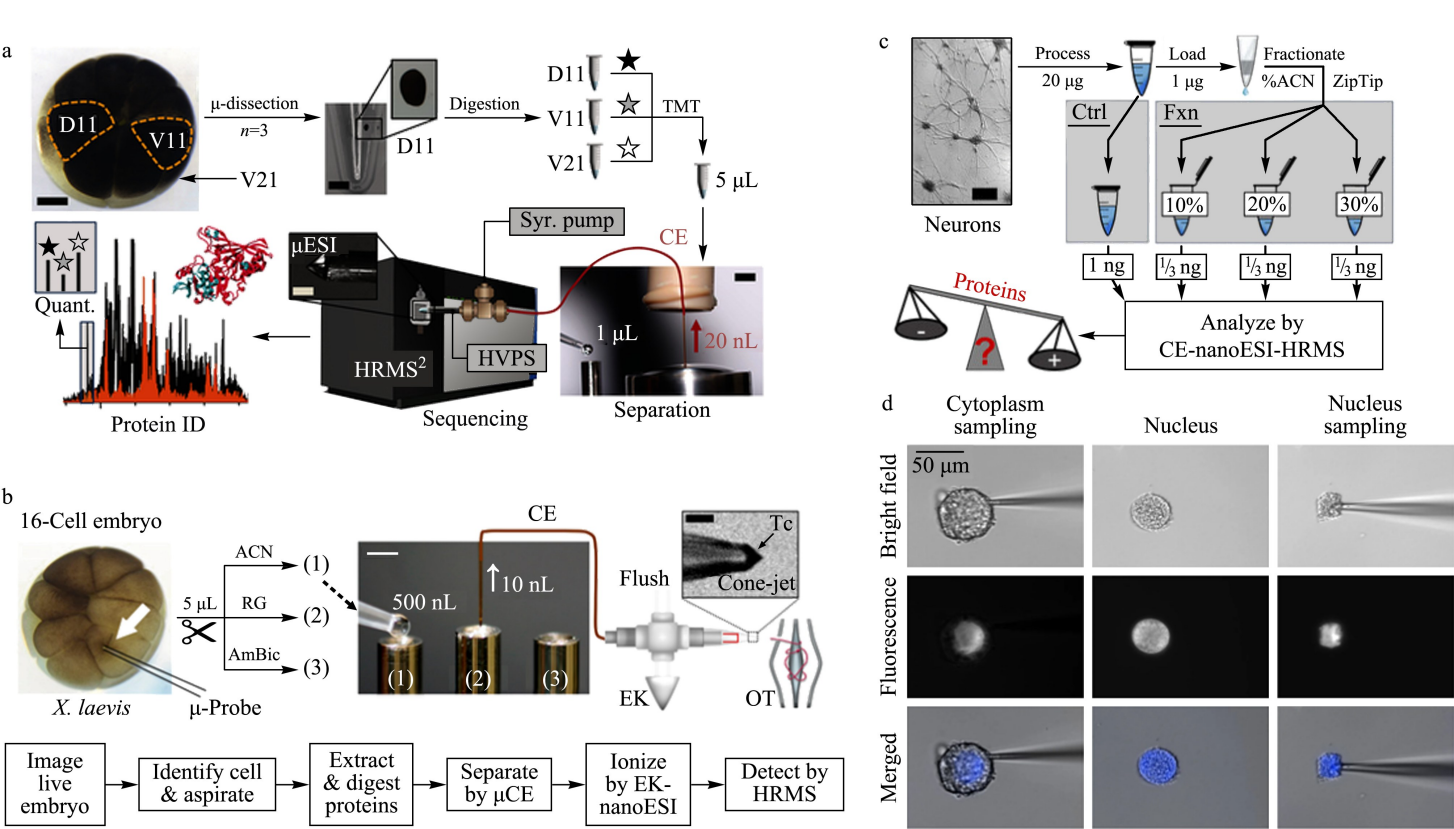
基于毛细管电泳的单细胞蛋白质研究^[19,22,24,28]^

如前所述,利用毛细管的微小管径实现单细胞内容物的提取以及转移,可避免单细胞有限体积的内容物由于容器和管路吸附等原因造成的样品损失,对于后续的蛋白质组学研究具有重要意义。Lee等^[27]^对生长在毛细管外壁的海兔神经元施加侧向刺激后,将释放的神经肽通过位于反射方向的填充毛细管柱(PEMC)进行收集,洗脱后利用基质辅助激光解吸附离子化质谱(MALDI-MS)检测刺激过程中单个神经元的神经肽含量的变化。将毛细管的直径进一步缩小至普通单细胞尺寸范围内(≤10 μm),可以实现亚细胞分辨率的直接检测。Zhang等^[28]^利用管径10 μm的毛细管对田螺特定神经元的细胞质以及细胞核进行先后取样(见图1d),进而利用离子淌度质谱(IMMS)发现了一种新的神经肽物种,揭示了神经元细胞内的区域表达异质性。

综上,基于CE-MS的单细胞分析应用主要集中在大体积细胞的蛋白质组研究上,其实验流程与常规的蛋白质组学基本一致。利用比单细胞尺寸更小的毛细管管径对亚细胞区域进行内容物的提取以及转移,是毛细管应用的一大特色。但CE与质谱接口的稳定性不足、CE方法重复性略差、电泳分离过程受pH值和温度等因素的影响,以及蛋白酶解物在电泳过程中的吸附造成样本损失等问题的存在,限制了CE-MS在单细胞蛋白质组学中的进一步应用。因此亟待开发更为实用、简便、易重复的方法。

## 2 基于液相色谱的分离

与毛细管电泳相比,液相色谱,尤其是纳升液相色谱(nanoLC)在单细胞蛋白质组学研究中的应用更为广泛^[14]^,这主要依赖于其良好的重现性、nL级进样量、较低的流速(nL/min)、较少的样品损失以及与nanoESI-MS的良好串联能力。其中,μm级内径的色谱柱提供了更高的分离能力,大大提高了被分析物的信号强度。nanoLC与高灵敏纳升电喷雾质谱的联用,被广泛应用于大体积的胚胎或生殖细胞,以及体细胞中。Sun等^[29]^利用纳升液相色谱的反相分离模式研究了非洲爪蟾早期胚胎的囊胚细胞,单次实验从16细胞分裂阶段的细胞中鉴定到了1400种蛋白质类别(见图2a),通过比较不同阶段蛋白质的表达情况,证实了随着分化程度的不断加深,囊胚细胞间的异质性逐渐增强。为了进一步提高肽段间的分离效果,Sun等^[29]^利用强阳离子交换色谱柱对样品进行预分离,然后利用超高效液相色谱-质谱(UPLC-MS)进行分析,借助8通道等重标签相对和绝对定量(iTRAQ)策略,一次实验比较了胚胎细胞的4个不同分化阶段(每阶段细胞利用两个通道标记),且鉴定蛋白质数目提升到4000种^[30]^。在体细胞分析方面,Slavov课题组^[31]^2018年发展了一种基于LC分离的单细胞蛋白质定性以及相对定量方法——单细胞蛋白组学质谱(SCoPE-MS)。该方法首先在显微镜下将单个Hela细胞挑选至玻璃管中,经过超声破碎、过夜酶解等蛋白质前处理步骤后,利用一系列串联质谱标签(tandem mass tag, TMT)标记技术对不同细胞间的蛋白质进行相对定量。标记后的不同细胞酶解产物混合后利用LC-MS/MS分析(见图2b)。工作中引入了“载体”(carriers)这一概念,即将上百个细胞按照单细胞的操作流程进行样品处理,利用单独的TMT通道进行标记,再与待测细胞混合后同时检测。载体的存在减少了单细胞样品由于表面吸附造成的损失,同时为质谱离子化过程提供足够的多肽,以获得足够的信息供后续多肽的鉴定。这一概念的提出为后续单细胞技术^[32]^的发展提供了重要参考。利用这一技术,他们研究了小鼠胚胎干细胞分化过程中蛋白质组层面的变化,并对不同分化时期的细胞进行了聚类分析,揭示了单细胞转录组以及蛋白质组方面的相似性以及差异性。然而,该方法的细胞分析通量依然有限,样品处理时间较长且蛋白质覆盖度不高。为了克服上述问题,该课题组^[33]^发展了第二代单细胞蛋白组学质谱技术,其主要改进在于通过在纯水中冷热交替实现细胞裂解;引入微孔板进行细胞样品处理从而提高通量;通过自动化操作提高分析效率。此外,结合数据驱动下的质谱参数优化(DO-MS)以及数据驱动下的保留时间归属(DART-ID)两种算法,实现了实验参数的交互优化,并提高了蛋白质归属的置信度,全方位地优化了单细胞蛋白组学质谱技术。利用该方法分析了在没有极化细胞因子的情况下,均质单核细胞分化成类巨噬细胞的过程,揭示了类巨噬细胞的蛋白质组连续变化的状态以及类巨噬细胞可能出现的异质性现象。得益于更少的样本损失及较高的分析通量,人们不断尝试在类似微孔板的微小体积内进行单细胞样品处理。张祥民课题组^[34]^于2015年报道了利用直接细胞进样、在线酶解、nanoLC-MS/MS分析的方法建立的第一代用于100个细胞分析的集成蛋白组分析装置(iPAD-100)。通过采用更细的色谱柱(22 μm)、更小直径的ESI喷针(3 μm)以及更高灵敏度的质谱,他们发展了iPAD-1方法(见图2c)^[35]^。结合毛细管的单细胞转移能力,在2 nL体积中实现细胞的管内裂解、蛋白质消化,通过后续的nanoLC-MS/MS分析,从单个Hela细胞中平均鉴定到126种蛋白质。

微流控技术由于具备以微米尺度空间对流体进行操控的特点,从而具有高集成性、高通量、自动化、廉价易得的优势^[36]^,近年来在单细胞分析技术中发展迅速。此外,纳升级液滴中存在明显的微滴加速现象^[37]^,也会提高蛋白质前处理的效率,因此基于液滴的微流控技术为单细胞蛋白质组学研究提供了新的、有效的工具。

**图2:**
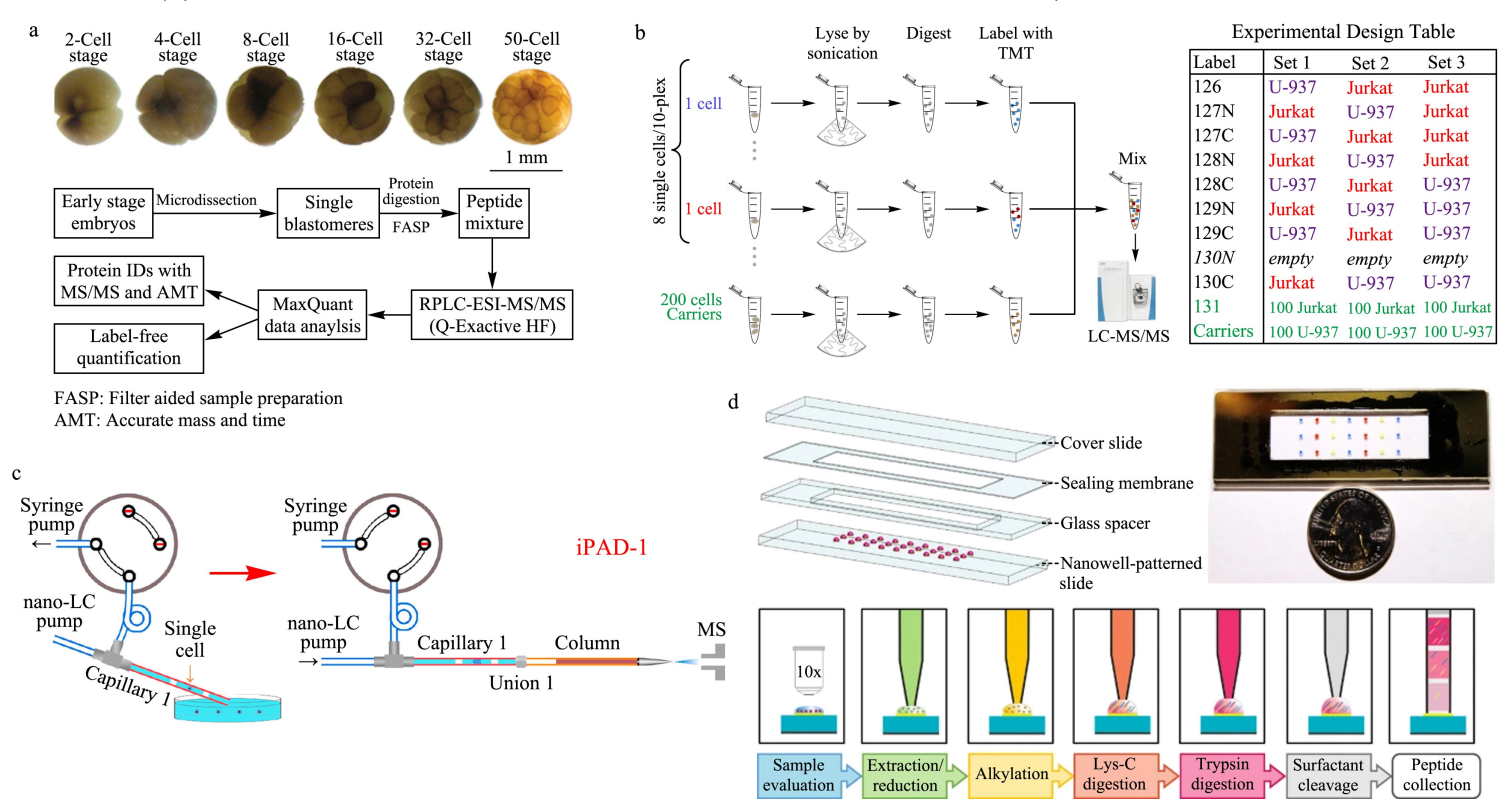
基于液相色谱的单细胞蛋白质研究^[29,31,35,39]^

2018年,Fang课题组^[38]^构建了纳升级空气-油界面的液滴负载芯片(oil-air droplet chip)。该芯片引入了油层,避免了有限体积液滴的挥发,使得细胞裂解、酶解等过程均可在一个液滴中进行,有效避免了单细胞样本的损失。样本处理后通过将每一个液滴吸入毛细管中,进入nanoLC-MS/MS进行分析。几乎在该技术出现的同时,Zhu等^[39]^也建立了一个纳升级反应器来实现少量细胞的蛋白质前处理,构建了一种痕量样品一体化处理平台,简称nanoPOTS(nanodroplet processing in one pot for trace samples)(见图2d)。其中每个液滴的体积仅有200 nL(面积为0.8 mm^2^),且利用石蜡膜进行密封可大大减少样品损失。利用该方法可从10~140个Hela细胞中鉴定到1500~3000种蛋白质。随后,他们对实验流程进行了进一步优化,包括引入流式细胞仪(FACS)进行单细胞的准确分选^[40]^,利用TMT技术进行相对定量^[32]^,使用更细管径的nanoLC柱进行分离,以及采用超高分辨率质谱进行数据采集^[41]^等。经过对nanoPOTS技术的多步优化和改进,最终在标记条件下实现单个Hela细胞中1400种蛋白质的鉴定,在无须标记条件下实现了大约360种蛋白质的鉴定,这也是迄今为止无标记条件下在单个体细胞中鉴定到的最多蛋白质数目。

总结上述工作可以发现,基于液相色谱的单细胞样本处理体积逐渐降至nL级别,将一系列蛋白质组学样品处理步骤整合在微小体积中,通过设置密封条件、减少洗涤步骤等方式来降低样品损失,进而结合nanoLC-MS进行分离和检测。然而,细胞裂解过程的充分与否,蛋白质前处理的步骤是否完整高效,以及是否对肽段进行标记等步骤仍然是影响蛋白质鉴定的数目与种类的重要因素。

## 3 无分离手段的直接检测

与借助CE和LC进行质谱前蛋白质分离相比,不经酶解和分离而直接进行质谱分析的方法由于待测物含量低、样品复杂程度提高,导致检测到的蛋白质种类十分有限。但其样品前处理步骤大大简化,蛋白质间的相对位置关系得以一定程度的保留,可提供目标分子的空间分布信息,为质谱成像提供了可能。其中最具代表性的技术有MALDI-MS以及二次离子质谱(SIMS)、无机质谱流式及其成像等。

MALDI-MS因其较高的空间分辨率,可以同时对组织中上百种小分子的种类及其分布进行分析^[42]^,因此在质谱成像中表现突出。Zavalin等^[43]^首先开发了真空透射式MALDI离子源,用于证实胰岛细胞内胰岛素的亚细胞定位,由于透射式的聚焦光路特点,该方法可实现1 μm的空间分辨率。然而,由于真空条件下细胞生理状态难以保持,该方法无法真实反映细胞内的蛋白质状态。而常压基质辅助激光解吸附离子化质谱(AP-MALDI-MS)可在接近细胞生理状态下对其进行分析,但是方法的分析灵敏度不足,使其在单细胞层面的应用面临挑战。2017年,Kompauer等^[44]^在AP-MALDI离子源的基础上进行了改进,通过激光共轴反射式的设计,以及使用较大数值孔径的聚焦物镜,获得了1.4 μm的激光空间分辨率,以及大于100000的质量分辨率,这也是目前AP-MALDI离子源所能达到的最高分辨率,并实现了亚细胞分辨率下肽段以及脂质代谢物的分析。由于传统小分子基质的干扰和离子抑制现象,利用MALDI离子源对细胞中小分子代谢物的鉴定相对困难。Comi等^[45]^结合液相微萃取手段,在MALDI-MS所提供的信息辅助下,对不同肽段含量的胰岛细胞进行分类,并采用萃取的方式对代谢物进行后续ESI检测,该方法避免了有机基质对代谢物检测的干扰,实现了代谢物与多肽的共同检测。为了进一步提高MALDI离子化效率,Niehaus等^[46]^在透射式MALDI成像装置中引入了红外激光诱导的后离子化过程,使得离子产率与灵敏度提高了几个数量级,空间分辨率达到600 nm,有望对一些低丰度肽段以及蛋白质实现亚细胞区域的成像。如前所述,MALDI基质会对待测分子检测产生抑制效应,因此如果将待测分子与基质在成像前进行分离,则会显著改善检测效果。Li等^[47]^利用纳秒激光触发的点击化学反应实现了局域空间(大约50 μm)内微电场和温度梯度的构建,以及蛋白质表面赖氨酸的标记,利用神经肽与基质小分子的迁移能力差异来实现两者的分离,从而提高小鼠脑组织中神经肽段检测灵敏度。该方法有望进一步拓展到单细胞水平。此外,Küster等^[48]^借助微流控技术实现MALDI靶板上的单液滴分散,利用MALDI-MS对单液滴中血管紧张肽的酶解产物进行了监控。如将该方法与单细胞分散进样相结合,可避免细胞间的交叉污染,有望用于单细胞的高通量分析。

SIMS自从诞生之初就以极高的空间分辨率以及三维动态成像能力在质谱成像领域独树一帜。纳米二次离子质谱(nanoSIMS)技术的横向分辨率甚至达到100 nm以内^[49]^,从空间分辨率的角度与单细胞甚至亚细胞水平相匹配。然而,有机分子和生物大分子的分析一直是SIMS的分析瓶颈。此外,SIMS技术对真空条件的要求高、同位素干扰明显以及定性能力差等因素的存在,也给单细胞蛋白质的研究带来了巨大阻碍^[50,51]^。截至目前,这方面的工作仍十分有限。现有方法多采用借助低背景同位素进行蛋白标记后检测的方式,研究通量大为降低。Vreja等^[52]^构建了一种F^19^同位素荧光双功能探针,通过非天然氨基酸插入以及点击化学反应将探针固定在靶蛋白表面,实现了SIMS以及荧光显微镜下单细胞双通道三维立体成像。

无机质谱流式(mass cytometry)在无机质谱免疫分析方法的基础上^[53]^,集成了传统流式细胞术的高通量,从原理上避免了荧光通道之间的干扰,大大提高了蛋白质检测数量和通量^[54]^。发展至今,已经在临床诊断^[4,55]^、药物筛选^[56]^等众多生命医学领域发挥了重要作用。无机质谱流式的原理是通过将稀土元素与抗体偶联形成探针,进而与细胞或者组织进行孵育,实现探针的识别。将分散后的单细胞以雾滴形式引入电感耦合等离子体-质谱(ICP-MS)进行检测。通过检测探针上标记的稀土元素的含量就可以实现单细胞中相应蛋白质的定量,从而得到不同单细胞的表达图谱。2017年,Bodenmiller课题组^[57]^应用无机质谱流式细胞仪分析了HEK 293T细胞中表皮生长因子受体信号网络及其动态变化与20种节点蛋白质丰度的关系,揭示了信号网络状态的丰度依赖性,并且证实了新的信号网络关系。此外,该课题组^[55]^还将其应用于固定组织的二维成像。利用探针标记组织后,通过直径1 μm的紫外激光实现标记组织表面稀土元素的解吸附,进而在气流带动下完成后续的ICP-MS检测。该方法同时定量了肿瘤组织中与乳腺癌相关的32种蛋白标志物的含量,在证实原有4种经典的乳腺癌疾病分型基础上,进一步提出了更多亚型的分类。值得注意的是,当时他们并没有整合空间信息来进行细胞分型,且病人样本数目较少。近期,他们对352个乳腺癌病人的组织进行了成像质谱流式的分析^[4]^,通过加入临近细胞簇的信息,创造性地提出了“细胞群落”的概念,并且据此产生的分型结果与长达15年的病人生存曲线结果有很好的对应关系。此外,他们后续结合了基因组^[58]^以及转录组^[59]^的视角对乳腺癌疾病给出了更为综合的多组学解释。该工作证实了单细胞蛋白质组学甚至多组学的研究对于肿瘤的精准诊断至关重要,无疑将推动单细胞技术在癌症精准医学方面的应用。

除了检测与抗体偶联的稀土元素含量外,利用一些有机质谱标签也可以实现蛋白质信号的转移甚至放大检测。相比无机质谱流式,有机质谱流式的方法不局限于稀土元素的种类以及纯度,可设计性更强,并且具有更好的信号放大能力,因此在未来有更大的发展空间。本课题组在这一领域开展了相关工作,例如Xu等^[60]^将质谱标签与靶标蛋白的抗体自组装在Au纳米颗粒表面,与细胞孵育实现蛋白质识别标记后,利用芯片喷雾实现多通道小分子标签的解离与检测,从而表征多种靶标蛋白的浓度。该方法的检出限可以低至zmol,已经比较接近单细胞的水平。进而Xu等^[61]^结合微流控芯片实现了单细胞排列,并利用nanoESI-HRMS,搭建了多维度有机质谱流式细胞分析平台。通过检测蛋白质标记质量标签和细胞内容物小分子,该平台创新性地实现了单细胞水平的6种蛋白质及84种代谢物的同时检测,并在多种肿瘤细胞分型和肿瘤耐药异质性研究中开展应用。

无分离方法直接检测的模式操作省去了繁杂的样品前处理步骤,操作相对简单,其中MALDI成像无需借助标记手段,但是截至目前空间分辨率比较有限,并且检测目标局限于部分代谢物以及肽段,而在成像前进行基质预分离可有效提高信号强度。而质谱流式以及SIMS需要借助标记的方法来进行信号转移,提供了高分辨的空间位置信息,其中无机质谱流式由于其多组学兼容能力,目前在精准医疗领域有较多的应用,而有机质谱流式由于其标签设计更为灵活且具有更强的信号放大能力,因此在单细胞分析方面正逐渐崭露头角。

综上,我们将基于不同分离方式的单细胞蛋白质质谱研究方法归纳于表1中,从表中可见不同的方法具有其各自的优势与局限。毛细管电泳的方法虽然成本较低,设备简单以及分离能力强,但是由于样品损失、细胞通量低,目前仍局限于较大体积细胞的蛋白质组学研究,未来可以尝试结合原位单细胞前处理流程以减少样品损失并提高细胞分析通量。与此同时,利用不同口径毛细管进行微区采样,为亚细胞分辨率的单细胞研究带来了便利和可能。基于液相色谱的方法,其蛋白质前处理步骤相对完整,但存在细胞通量相对较低和蛋白质的覆盖度低等问题。未来可以从发展低样本吸附的方法,开发高通量单细胞分离技术,借助标记技术定量以及利用更高性能的质谱仪器来实现分析结果的进一步优化。对于无分离模式下的质谱直接分析方法,目前仍无法实现蛋白质大分子的直接检测,但利用免疫标记探针和质谱技术可实现单细胞上多个蛋白质的检测。后续工作中,如何提高蛋白质的分析通量、发展新型稳定蛋白质标记技术以及信号输出技术将是单细胞质谱方法发展的关键。可见,发展高通量的细胞分析策略,高蛋白覆盖度以及具有空间分辨的蛋白质鉴定技术将是该领域面临的挑战和进一步发展的方向。

**表1:** 代表性的单细胞蛋白质组学研究方法

Separation mean	Method	Throughput (cell numbers/ run)	Identified protein number^1)^	Sensitivity	Application
CE based	microdissection^[22]^	1	1630 (label)^2)^	25 amol	differentiation process and subcellular asymme-
	macrosampling^[19]^	1	341	700 zmol	try of Xenopus embryo, zebrafish embryos
	RP pre-fractionation^[24]^	1	141	260 zmol	mouse hippocampal neurons proteome
LC based	microdissection^[29]^	1	1500	-	Xenopus embryo differentiation process and
	microdissection with iTRAQ strategy^[30]^	8	~4000 (label)	-	early development
	SCoPE^[31]^	~190	767 (label)	zmol	differentiation of mouse embryonic stem cells
	SCoPE2^[33]^	>2000	>1000 (label)	zmol	differentiation mechanism of homogeneous monocytes
	OAD chip^[38]^	1	51	-	Hela cell proteome
	iPAD-1^[35]^	1	126	1.7 zmol	histone profiling in Hela cell cycle
	nanoPOTs^[32]^	72	1400 (label)	amol	classification of Epithelial cells and immune cells in mice
	enhanced nanoPOTs^[41]^	72	362	<amol	Hela cell proteome
Direct detection	AP-MALDI^[44]^	~10^4^-10^5^	220 peptides	-	mice brain tissue imaging
based	SIMS^[52]^	~10^3^-10^4^	<10	-	three-dimensional imaging of cells
	mass cytometry^[57]^	~10^5^-10^6^	40	zmol	cellular signaling network changes
	mass cytometry imaging^[4]^	~10^4^-10^5^	40	zmol	breast cancer subtyping

1) Identified protein numbers refer to single measurement result. 2) Label means peptide labelling technology is employed in corresponding method. iTRAQ: isobaric tags for relative and absolute quantitation; ScoPE: single cell ProtEomics; OAD: oil-air-droplet; nanoPOTS: nanodroplet processing in one pot for trace samples; AP-MALDI: atmospheric pressure-matrix-assisted laser desorption/ionization mass spectrometry; SIMS: secondary ion mass spectrometry; -: no clear data.

## 4 总结与展望

通过对以上工作的梳理与思考,我们对单细胞蛋白质组学研究进行了展望。

首先,从多组学的角度对单细胞进行综合分析将是未来单细胞技术的必由之路,驱动力来自于人们越来越需要对细胞进行综合分析以及整体研判来解释愈加复杂的生物学问题,进而对疾病治疗给出更为精确的方案。《自然》出版社将“单细胞多组学技术”评为“2019年度技术”,一些学者也对此表示了充分的赞同与肯定^[62,63,64]^。目前,在单细胞转录组领域,将基因组以及转录组进行结合的方法并不少见,而将转录组与蛋白质结合的代表性例子只有单细胞RNA测序方法CITE-seq^[65]^以及REAP-seq^[66]^,这两种方法都使用了寡聚核苷酸链偶联的抗体来实现蛋白质信号与转录组测序的整合,但是蛋白鉴定数量不超过80种。由于种类多样组成复杂,单细胞蛋白质组学与代谢组学的整合目前还未见报道。考虑到质谱强大的代谢物鉴定能力,未来基于质谱的多组学技术具有非常大的研究潜力。总之,发展单细胞多组学技术,解决蛋白质信号如何与其他组学信息兼容问题,并最终实现同时检测与数据分析,是研究人员今后一段时间内所要面临的巨大挑战。

其次,微流控技术的天然优势将为单细胞分析带来高通量以及自动化的可能,高通量大大缩减了样品处理时间,而自动化则减少了由于人为操作所带来的少量样品处理的可能误差^[67]^,这在处理大量细胞的过程中尤为重要。目前基于微流控技术的单细胞蛋白质组学方法多基于静态液滴形式,因此在通量方面仍有很大的提高空间,利用微流控芯片强大的可操控性,在流路中对单细胞复杂的微环境进行模拟以及实时改变外界刺激将会更真实地反应体内单细胞的生存环境以及变化过程^[68]^,对于疾病的诊断与了解将会更加准确与深入。与此同时,发展微流控与质谱的在线联用技术,无疑将使细胞通量以及灵敏度等得到进一步提升。然而如何将不连续的蛋白质前处理流程与连续的检测相结合,充分发挥在线联用高通量优势的同时,尽可能减少样品损失将是必须解决的问题。此外,由于质谱是目前单细胞蛋白质组学研究的必要工具,发展高性能的质谱仪器,建立新的质谱检测方法以及与之配套的蛋白质鉴定软件等都将是领域关注的重点。

当然,除了方法本身的纵向发展外,与其他技术相结合的“横向发展”也十分重要。例如在现有研究方法基础上,进行特定亚细胞区域的蛋白质组学、相互作用蛋白质组学以及某种特定翻译后修饰类型的蛋白质组学的研究等。这将有助于诸多生化过程以及致病机理的全新认识与发现,Slavov^[69]^在相关综述中也表达了类似想法。

综上,随着基于单细胞蛋白质组学质谱研究方法的不断发展,单细胞分析方法的灵敏度、蛋白质的覆盖度、细胞通量、空间分辨率以及多组学的兼容能力会不断突破我们的认知极限。更重要的是,单细胞分析在临床诊断、疾病分型以及细胞发展机制这些重大生命科学领域方面的应用将会越来越广泛。

## References

[1] Barabasi A L, Oltvai Z N. Nat Rev Genet, 2004,5(2):101 14735121 10.1038/nrg1272

[2] Klosin A, Oltsch F, Harmon T, et al. Science, 2020,367(6476):464 31974256 10.1126/science.aav6691

[3] Sun X X, Yu Q. Acta Pharmacol Sin, 2015,36(10):1219 26388155 10.1038/aps.2015.92 PMC4648179

[4] Jackson H W, Fischer J R, Zanotelli V R T, et al. Nature, 2020,578(7796):615 31959985 10.1038/s41586-019-1876-x

[5] Stewart C A, Gay C M, Xi Y, et al. Nat Cancer, 2020,1(4):423 33521652 10.1038/s43018-019-0020-z PMC7842382

[6] Labib M, Kelley S O. Nat Rev Chem, 2020,4(3):143 10.1038/s41570-020-0162-7 37128021

[7] Dahlberg P D, Saurabh S, Sartor A M, et al. Proc Natl Acad Sci U S A, 2020,117(25):13937 32513734 10.1073/pnas.2001849117 PMC7321984

[8] Li Y, Qian Z, Ma L, et al. Nat Commun, 2016,7(1):12906 27686409 10.1038/ncomms12906 PMC5056435

[9] Wittenberg N, Maxson M, Eves D, et al. Frontiers in Neuroengineering Electrochemistry at the Cell Membrane/Solution Interface. Michael A C, Borland L M, transl. Boca Raton: CRC Press/Taylor & Francis, 2007 21204382

[10] Chen X, Dong T, Wei X, et al. Biosens Bioelectron, 2019,142:111453 31295711 10.1016/j.bios.2019.111453

[11] Tonello S, Stradolini F, Abate G, et al. Sci Rep, 2019,9(1):17347 31758050 10.1038/s41598-019-53994-6 PMC6874615

[12] Liu L, Chen D, Wang J, et al. Cells, 2020,9(5):1271

[13] Yang L, George J, Wang J. Proteomics, 2020; 20(13):1900226 10.1002/pmic.201900226 PMC7225074 31729152

[14] Shao X, Weng L X, Gao M X, et al. TrAC-Trends Anal Chem, 2019,120:115666

[15] Pham T, Tyagi A, Wang Y S, et al. Wiley Interdiscip Rev Syst Biol Med, 2020. doi: 10.1002/wsbm.1503

[16] Specht H, Slavov N. J Proteome Res, 2018,17(8):2565 29945450 10.1021/acs.jproteome.8b00257 PMC6089608

[17] Delaney K, Sauer C S, Vu N Q, et al. Molecules, 2018,24(1):42 10.3390/molecules24010042 PMC6337428 30583525

[18] Lombard-Banek C, Choi S B, Nemes P. Single-Cell Proteomics in Complex Tissues using Microprobe Capillary Electrophoresis Mass Spectrometry. Allbritton N L, Kovarik M L, transl. New York: Academic Press, 2019: 263 10.1016/bs.mie.2019.07.001 PMC7397975 31668233

[19] Lombard-Banek C, Moody S A, Manzini M C, et al. Anal Chem, 2019,91(7):4797 30827088 10.1021/acs.analchem.9b00345 PMC6688183

[20] Sun L, Hebert A S, Yan X, et al. Angew Chem Int Ed Engl, 2014,53(50):13931 25346227 10.1002/anie.201409075 PMC4392885

[21] Sun L, Zhu G, Mou S, et al. J Chromatogr A, 2014,1359:303 25082526 10.1016/j.chroma.2014.07.024 PMC4141985

[22] Lombard-Banek C, Moody S A, Nemes P. Angew Chem Int Ed, 2016,55(7):2454 10.1002/anie.201510411 PMC4755155 26756663

[23] Flachsova M, Sindelka R, Kubista M. Sci Rep, 2013,3:2278 23880666 10.1038/srep02278 PMC3721081

[24] Choi S B, Lombard-Banek C, Muñoz-Llancao P, et al. J Am Soc Mass Spectrom, 2018,29(5):913 29147852 10.1007/s13361-017-1838-1

[25] Geng X, Shi M, Ning H, et al. Talanta, 2018,182:279 29501153 10.1016/j.talanta.2018.01.076

[26] Chen D, Fan F, Zhao X, et al. Anal Chem, 2016,88(4):2466 26810843 10.1021/acs.analchem.5b04645

[27] Lee C Y, Fan Y, Rubakhin S S, et al. Sci Rep, 2016,6(1):26940 27245782 10.1038/srep26940 PMC4887886

[28] Zhang L, Khattar N, Kemenes I, et al. Sci Rep, 2018,8(1):12227 30111831 10.1038/s41598-018-29704-z PMC6093924

[29] Sun L, Dubiak K M, Peuchen E H, et al. Anal Chem, 2016,88(13):6653 27314579 10.1021/acs.analchem.6b01921 PMC4940028

[30] Sun L, Bertke M M, Champion M M, et al. Sci Rep, 2014,4(1):4365 24626130 10.1038/srep04365 PMC3953746

[31] Budnik B, Levy E, Harmange G, et al. Genome Biol, 2018,19(1):161 30343672 10.1186/s13059-018-1547-5 PMC6196420

[32] Dou M, Clair G, Tsai C F, et al. Anal Chem, 2019,91(20):13119 31509397 10.1021/acs.analchem.9b03349 PMC7192326

[33] Specht H, Emmott E, Petelski A A, et al. bioRxiv, 2019: 665307. doi: 10.1101/665307

[34] Chen Q, Yan G, Gao M, et al. Anal Chem, 2015,87(13):6674 26061007 10.1021/acs.analchem.5b00808

[35] Shao X, Wang X, Guan S, et al. Anal Chem, 2018,90(23):14003 30375851 10.1021/acs.analchem.8b03692

[36] Liu Y, Chen X, Zhang Y, et al. Analyst, 2019,144(3):846 30351310 10.1039/c8an01503a

[37] Wei Z, Li Y, Cooks R G, et al. Annu Rev Phys Chem, 2020,71(1):31 32312193 10.1146/annurev-physchem-121319-110654

[38] Li Z Y, Huang M, Wang X K, et al. Anal Chem, 2018,90(8):5430 29551058 10.1021/acs.analchem.8b00661

[39] Zhu Y, Piehowski P D, Zhao R, et al. Nat Commun, 2018,9(1):882 29491378 10.1038/s41467-018-03367-w PMC5830451

[40] Zhu Y, Clair G, Chrisler W B, et al. Angew Chem Int Ed, 2018,57(38):12370 10.1002/anie.201802843 PMC6261339 29797682

[41] Cong Y, Liang Y, Motamedchaboki K, et al. Anal Chem, 2020,92(3):2665 31913019 10.1021/acs.analchem.9b04631 PMC7550239

[42] Eriksson C, Masaki N, Yao I, et al. Mass Spectrom (Tokyo), 2013,2:S0022 24349941 10.5702/massspectrometry.S0022 PMC3809716

[43] Zavalin A, Todd E M, Rawhouser P D, et al. J Mass Spectrom, 2012,47(11):1473 23147824 10.1002/jms.3108

[44] Kompauer M, Heiles S, Spengler B. Nat Methods, 2017,14(1):90 27842060 10.1038/nmeth.4071

[45] Comi T J, Makurath M A, Philip M C, et al. Anal Chem, 2017,89(14):7765 28636327 10.1021/acs.analchem.7b01782 PMC5518278

[46] Niehaus M, Soltwisch J, Belov M E, et al. Nat Methods, 2019,16(9):925 31451764 10.1038/s41592-019-0536-2

[47] Li G, Ma F, Cao Q, et al. Nat Commun, 2019,10(1):4697 31619683 10.1038/s41467-019-12548-0 PMC6795811

[48] Küster S K, Fagerer S R, Verboket P E, et al. Anal Chem, 2013,85(3):1285 23289755 10.1021/ac3033189

[49] Gamble L J, Anderton C R. Micros Today, 2016,24(2):24 27660591 10.1017/S1551929516000018 PMC5028133

[50] Nuñez J, Renslow R, Cliff J B, et al. Biointerphases, 2017, 13(3):03B301 10.1116/1.4993628 28954518

[51] Agüi-Gonzalez P, Jähne S, Phan N T N. J Anal At Spectrom, 2019,34(7):1355

[52] Vreja I C, Kabatas S, Saka S K, et al. Angew Chem Int Ed, 2015,54(19):5784 10.1002/anie.201411692 PMC4471591 25783034

[53] Bandura D R, Baranov V I, Ornatsky O I, et al. Anal Chem, 2009,81(16):6813 19601617 10.1021/ac901049w

[54] Spitzer Matthewh, Nolan Garryp. Cell, 2016,165(4):780 27153492 10.1016/j.cell.2016.04.019 PMC4860251

[55] Giesen C, Wang H A O, Schapiro D, et al. Nat Methods, 2014,11(4):417 24584193 10.1038/nmeth.2869

[56] Atkuri K R, Stevens J C, Neubert H. Drug Metab Dispos, 2015,43(2):227 25349123 10.1124/dmd.114.060798

[57] Lun X K, Zanotelli V R T, Wade J D, et al. Nat Biotechnol, 2017,35(2):164 28092656 10.1038/nbt.3770 PMC5617104

[58] Arnol D, Schapiro D, Bodenmiller B, et al. Cell Rep, 2019,29(1):202 31577949 10.1016/j.celrep.2019.08.077 PMC6899515

[59] Schulz D, Zanotelli V R T, Fischer J R, et al. Cell Syst, 2018,6(4):531 29698648 10.1016/j.cels.2018.04.004 PMC5929910

[60] Xu S, Ma W, Bai Y, et al. J Am Chem Soc, 2019,141(1):72 30565930 10.1021/jacs.8b10853

[61] Xu S, Liu M, Bai Y, et al. Angew Chem Int Ed, 2020, doi: 10.1002/anie.202009682

[62] Zhu C, Preissl S, Ren B. Nat Methods, 2020,17(1):11 31907462 10.1038/s41592-019-0691-5

[63] Marx V. Nat Methods, 2019,16(9):809 31406385 10.1038/s41592-019-0540-6

[64] Schier A F. Nat Methods, 2020,17(1):17 31907464 10.1038/s41592-019-0693-3

[65] Stoeckius M, Hafemeister C, Stephenson W, et al. Nat Methods, 2017,14(9):865 28759029 10.1038/nmeth.4380 PMC5669064

[66] Peterson V M, Zhang K X, Kumar N, et al. Nat Biotechnol, 2017,35(10):936 28854175 10.1038/nbt.3973

[67] Hosic S, Murthy S K, Koppes A N. Anal Chem, 2016,88(1):354 26567589 10.1021/acs.analchem.5b04077 PMC4809371

[68] Michna R, Gadde M, Ozkan A, et al. Biotechnol Bioeng, 2018,115(11):2793 29940072 10.1002/bit.26778 PMC6261298

[69] Slavov N. Science, 2020,367(6477):512 32001644 10.1126/science.aaz6695 PMC7029782

